# The impact of stereotype threat on endogenous poverty-elimination dynamics in generationally poor individuals

**DOI:** 10.3389/fpsyg.2023.1174614

**Published:** 2023-03-30

**Authors:** Na Wu, Anguo Fu, Yangxiong Liu, Tong Yue, Jibo Li, Xiaogang Wang, Xiting Huang

**Affiliations:** ^1^Research Center of Psychology and Social Development, Faculty of Psychology, Southwest University, Chongqing, China; ^2^Management School, Hainan University, Haikou, China; ^3^Guangdong Provincial Key Laboratory of Development and Education for Special Needs Children, Department of Psychology, Lingnan Normal University, Zhanjiang, China; ^4^School of Education and Psychology, Southwest Minzu University, Chengdu, China

**Keywords:** endogenous poverty-elimination dynamics, stereotype threat, generationally poor individuals, self-affirmation, intervention technique

## Abstract

**Introduction:**

The study examines the impact of stereotype threat on generationally poor individuals and its effect on achievement motivation. It also explores the extent to which self-affirmation has an intervention effect on the negative impact of stereotype threat.

**Methods and results:**

In Study 1, statements that contained negative stereotypes were used to elicit stereotype threat in generationally poor individuals; the results show that stereotype threat reduced the performance of generationally poor individuals in a mental-rotation task. Study 2 used a questionnaire to measure the endogenous dynamics of generationally poor individuals attempting to escape poverty after experiencing stereotype threat; participants in the stereotype-threat group showed lower-level endogenous poverty-elimination dynamics than those in the control group. In Study 3, a self-affirmation intervention was administered to the stereotype-threat group after the stereotype threat was induced. Participants in the self-affirmation group were shown to have higher-level endogenous poverty-elimination dynamics than those in the control group.

**Discussion:**

These findings confirm the negative effect of stereotype threat on endogenous poverty-elimination dynamics and verify the effectiveness of self-affirmation in mitigating the negative effects of stereotype threat.

## 1. Introduction

Franklin once said, “Poverty itself is not terrible, what is terrible is the thought that one is destined to be poor or that one must die of poverty.” External factors act through internal factors. Endogenous poverty-elimination dynamics constitute a key psychological resource that can be indispensable in alleviating poverty among poor individuals. In the context of poverty alleviation in China, it is apparent that “endogenous dynamics” differ from the “intrinsic motivation” referred to in self-determination theory in the Western psychological context (Deci and Ryan, 1985). Self-determination theory suggests that three basic psychological needs—autonomy, competence, and relatedness—are essential for an individual’s psychological growth, internalization, and psychological wellbeing (Ryan and Deci, 2000, 2017). Only when an organizational environment satisfies these three basic psychological needs can individuals experience a sense of willingness, will, and choice in their work activities; enhance or maintain intrinsic motivation; and produce more consistently productive behavioral outcomes, while enhancing their physical and mental health (Gagné and Deci, 2005). Many poor people in China have lived for generations in isolated, slow-changing, and poverty-stricken areas. Individuals in such environments have little contact with the outside world and little awareness of their own poverty, which makes it difficult for them to generate enough autonomous (bottom-up) intrinsic motivation to escape poverty. According to self-determination theory, the pursuit of autonomy, competence, and relatedness derives from an intrinsic, fundamental aspect of human nature, unrelated to environmental influences or cultural upbringing. Specifically, intrinsic motivation in Western psychology is primarily a product of Western European and North American cultures, based on the specific assumptions and beliefs of Western researchers about human nature (Markus and Kitayama, 1998). These highly individualistic beliefs about independence, autonomy, and being separate from society are based on Judeo-Christian ideas regarding “good people.” By contrast, in the context of China, poverty elimination is dominated by the central government’s top-down poverty-reduction approach, which is based on collective power and wisdom. Thus, endogenous poverty-elimination dynamics, as a concept, is well-adapted to Chinese culture (Fu and Fu, 2020), which is typically associated with collective beliefs about individual morality, rights, and responsibilities (based on the collectivist beliefs of Confucianism).

Endogenous poverty-elimination dynamics can be categorized as a type of personality dynamics. It is important to note that an individual’s ability to overcome poverty is not solely determined by a specific psychological trait, but also by the overall endogenous personality dynamics that motivates them. These endogenous dynamics serve as the driving force behind the behavior of impoverished individuals and consist of various psychological factors, such as values, self-concept, and behavioral tendencies (Fu et al., 2020). Through a series of studies conducted in the Chinese context, researchers have found that the “endogenous dynamics” of poverty alleviation are the individual’s tendency to pursue poverty alleviation as a goal through personal striving, guided by a core value system (Fu and Fu, 2020; Fu et al., 2020). These endogenous dynamics consist of three internal factors, which are wrapped in layers that include core values, self-perception, and a behavioral tendency toward escaping poverty. In academic discourse in English-speaking countries, the term “Generationally Poor” usually refers to a condition of intergenerational poverty. This demographic is characterized by the persistent poverty across several successive generations within a family, which creates significant challenges in breaking free from the vicious cycle of poverty (Graves, 2017). Similarly, in Chinese academic circles, the term “Generationally Poor Individuals” (Fu et al., 2020) is frequently used to describe people whose families have experienced poverty for three or more generations in research studies. They are characterized by low-level endogenous dynamics toward escaping poverty. Due to their long-term economic disadvantages, poor individuals are often labeled with negative stereotypes that are derogatory and insulting, such as “lazy” and “short-sighted.” These stereotypes expose generationally poor people to rejection, discrimination, and ridicule. Although the resulting stereotype threat can cause intangible harm, few studies have addressed the stereotype threat experienced by this vulnerable group of generationally poor individuals. As a result, little is known about the impact of such situations.

One negative consequence of stereotype threat is a decreased level of motivation among individuals in the threatened domain (Cadaret et al., 2017). Endogenous poverty-elimination dynamics, as a personal dynamic associated with escaping poverty, is very similar to motivation. Thus, stereotype threat is likely to be its antecedent. No previous researchers have explored the antecedents of endogenous poverty-elimination dynamics among generationally poor individuals from a stereotype-threat perspective. Thus, the first question addressed in this study is: Does stereotype threat reduce endogenous poverty-elimination dynamics among generationally poor individuals? It is also worth asking which interventions can be used in this context. Self-affirmation has been proposed as an effective intervention for mitigating stereotype threat. First, self-affirmation can effectively mitigate the ego depletion caused by stereotype threat. Second, it can reduce the negative impact of stereotype threat on self-integrity by enabling people to view themselves and their resources in a more holistic way (Cohen and Sherman, 2014). As self-affirmation has been widely shown to mitigate the negative effects of stereotype threat (Sherman et al., 2013; Kinias and Sim, 2016; Hadden et al., 2020), the present study has selected this intervention.

In summary, this study investigates the impact of stereotype threat on endogenous poverty-elimination dynamics and the intervention effect of self-affirmation; it does this by inducing stereotype threat among individuals with generational poverty.

### 1.1. The impact of stereotype threat on endogenous poverty-elimination dynamics

Stereotype threat is a negative experiential process that occurs when an individual or group perceives a negative stereotype and is afraid of conforming to that stereotype (Steele and Aronson, 1995). It is essentially a pervasive self-evaluative threat, which varies in its nature and degree, depending on the group or situation involved. The threatened individual does not have to believe in the negative stereotype to experience threat; however, once created, negative stereotypes can be difficult to eliminate through disproof (Steele, 1997). In previous research, individuals threatened by negative stereotypes have shown reduced levels of motivation in the corresponding field. For example, stereotype threat can reduce women’s motivation to major in engineering or pursue related careers (Cadaret et al., 2017). Among children, gender stereotypes reduce girls’ motivation to study STEM subjects (science, technology, engineering, or mathematics) (Master, 2021). According to Rahn et al. (2021), age stereotypes affect the social motivation of older adults, making them more likely to adopt social-avoidance strategies and potentially impacting their success in the work domain. In previous research, mental-rotation tasks have been used to examine stereotype-threat activation (Dunst et al., 2013), with some studies focusing on working memory as the explanatory mechanism (Rydell et al., 2010). Combined with the integrated process model of stereotype threat proposed by Schmader et al. (2008), stereotype-threat-related information has been shown to cause individuals living with generational poverty to feel pressured. In response, they monitor their behavioral performance consciously and suppress their negative emotions. As these processes take up a large amount of working memory, inadequate working-memory resources are left to deal with complex cognitive processes, causing poorer performance in mental-rotation tasks. Because mental-rotation task rely so heavily on working-memory capacity, the present study has used a mental-rotation task to examine the stereotype-threat effect.

Fu et al. (2019) observed that individuals who have experienced intergenerational poverty, defined as their families being in poverty for three or more generations, are subject to negative stereotypes related to their appearance, personality, behavioral habits, abilities, and interpersonal communication. Compared to other socio-economically disadvantaged groups, intergenerational poverty individuals have developed a more deeply entrenched poverty culture, which includes a heightened risk of stereotypes, due to the intergenerational transmission and accumulation of poverty. This seriously hinders their endogenous poverty-elimination dynamics. There are three reasons for this: first, stereotype threat is a type of social threat (Hirschberger et al., 2016) that can negatively affect individual self-efficacy (Cadaret et al., 2017), which reflects the extent to which individuals believe they can do something (Bundura, 1977). Lower levels of self-efficacy decrease the extent to which individuals are motivated to succeed, which then reduces their endogenous poverty-elimination dynamics. Second, stereotype threat may take up too much working memory, which could otherwise be used to deal with problems (Beilock et al., 2007); this can make it difficult for individuals to maintain their motivation levels. Third, stereotype threat can cause individuals to disidentify with a particular domain (Woodcock et al., 2012), reducing their motivation levels in that domain. Since endogenous poverty-elimination dynamics in China and intrinsic motivation in the West are both personality dynamics, stereotype threat is likely to have a negative impact on both, including endogenous poverty-elimination dynamics in generationally poor individuals.

### 1.2. The impact of self-affirmation on stereotype threat

Steele (1999) defined self-affirmation as a way in which individuals can affirm their self-worth in areas unrelated to stereotype threat in order to maintain self-integrity, i.e., the belief in themselves as good people. Self-affirmation reduces the defensive responses that people use to fend off threats. From the perspective of experimental manipulation, self-affirmation approaches can be categorized as positive feedback versus affirmation of core self-worth. They can also be categorized by content, as individual versus group forms of self-affirmation. In a meta-analysis of stereotype-threat interventions, Liu et al. (2021) defined self-affirmation as an approach based on enhancing individual psychological resilience.

Self-affirmation is now widely used to counteract the effects of stereotype threat in areas such as STEM (Hadden et al., 2020), gender (Kinias and Sim, 2016), and race (Sherman et al., 2013). Hall et al. (2014) used self-affirmation to help low-income groups affected by stigmatizing labels, such as incompetence. They found that self-affirmation (achieved by verbalizing personal experiences) made the subjects feel successful and proud, reducing the stigma of poverty. Individuals who engaged in self-affirmation demonstrated better executive control than those who did not. The present study uses self-affirmation to mitigate the negative effects of stereotype threat on individuals living with generational poverty. The reasons for this are 2-fold: first, self-affirmation enables people to appreciate themselves and their resources while coping with the effects of stereotype threat. By allowing threatened individuals to view themselves more holistically and objectively, it diminishes the negative effects of threat on self-integrity (Cohen and Sherman, 2014). Self-affirmation also helps to reduce defensive responses to threats, such as denial, thus compensating to some extent for the ego depletion caused by stereotype threat (Badea and Sherman, 2019). The present study has therefore used self-affirmation as an intervention to mitigate the negative effects of stereotype threat.

In summary, generationally poor individuals are vulnerable to negative stereotypes imposed by other individuals and society in their daily lives. Those who are threatened by negative stereotypes tend to show lower levels of motivation in related areas and to have lower endogenous poverty-elimination dynamics than non-poor individuals. We therefore hypothesize that stereotype threat is one reason for the reduced endogenous poverty-elimination dynamics found among poor individuals. China researchers have also shown that stereotype threat has a higher level of ecological validity than implicit association tests in explaining bias because the terms used to convey negative stereotypes are more common in everyday life settings (Yu and Liang, 2005). The present study begins by using sentences containing negative-stereotype-related words to elicit stereotype threat in generationally poor individuals. It also uses a mental-rotation task that is commonly used to test stereotype threat, with changing response times in the mental-rotation task used to test induction validity. The study further explores the effect of stereotype threat on endogenous poverty-elimination dynamics in this group of individuals, while investigating the intervention effect of self-affirmation.

## 2. Study 1 inducing stereotype threat in generationally poor individuals

### 2.1. Purpose of the study

This study uses a mental-rotation task to elicit and test stereotype threat in generationally poor individuals. The experiment has the following two aims: first, to select vocabulary related to negative stereotypes of generationally poor individuals from the literature on endogenous poverty-elimination dynamics in poor individuals, and then to prepare statements to use in stimulus materials to elicit stereotype threat; and second, to use a mental-rotation task to test stereotype-threat elicitation and its effects.

### 2.2. Material selection

Ten terms related to negative stereotypes of generationally poor individuals were selected from an article by Fu et al. (2020) on endogenous poverty-elimination dynamics. The negative stereotypes were as follows: being passive and afraid of difficulty, being lazy and unable to cope, have low self-esteem and negativity, being short-sighted, drifting along, being speculative and following others blindly, being weak-willed, being poor at communication, being snobbish or jealous, and lacking commitment. The scope of endogenous poverty-elimination dynamics included three main categories: values, self-perception, and behavioral tendencies to escape poverty.

The validity of these ten descriptive terms was rated using a 7-point scale (1 = very negative–7 = very positive). An online questionnaire was distributed on the Credamo platform and 50 valid questionnaires were obtained. The questionnaire data were processed and the mean score of each term was compared with the median value of four; a one-sample *t*-test was conducted and the test results are shown in Table 1. The verification process found that all mean scores were significantly smaller than the median value of four; in other words, all 10 terms had a significant degree of negativity. These results indicate that the selected materials could be used to induce the threat of negative stereotypes among individuals living with generational poverty.

**TABLE 1:** Validity scores for negative stereotypes.

	M	SD	t	Cohen’s d
Afraid of difficulty and passive	2.05	0.72	–18.95**	2.73
Too lazy to cope	1.86	0.73	–21.52**	3.06
Negative, with low self-esteem	2.72	0.73	–12.96**	1.86
Short-sighted	1.90	0.77	–20.06**	2.83
Drifting along	1.95	0.81	–18.31**	2.61
Speculative and following others blindly	2.62	0.68	–15.01**	2.13
Weak-willed	2.16	0.78	–17.42**	2.45
Poor at communication	3.06	0.65	–10.17**	1.45
Snobbish and jealous	1.64	0.69	–25.26**	3.61
Lacking commitment	2.46	0.65	–16.32**	2.33

**p < 0.01.

The descriptive terms above were incorporated into complete declarative sentences as stimuli in the follow-up experiment, with each sentence including two elements: group affiliation and negative stereotypes, e.g., “People from poor families (group affiliation) are short-sighted (negative stereotype).” A total of 10 statements were obtained as material for the stereotype-threat group. The control group was matched with neutral statements of the same length, e.g., “Milliseconds constitute a common unit of time.”

### 2.3. Research methods

#### 2.3.1. Participants

The participants selected for this study all came from families in which three or more generations had lived in poverty. They were identified through the subsidies they received from government departments in Hainan Province, using the following evaluation criteria: (1) each family had a poverty certificate issued by a government department; (2) each family had been poor for three generations or more; (3) each family income was lower than the local minimum living standard in 2022.

Study 1 selected 120 generationally poor individuals as study participants and invited them to participate in the trial after obtaining their informed consent. Of these, 49 were males with a mean age of 33.69 years (SD = 1.33) and 71 were females with a mean age of 37.32 years (SD = 1.19). All participants were randomly assigned to either the stereotype-threat group (26 males and 34 females) or the control group (23 males and 37 females). The sample size was estimated using G*power 3.1.9.7. At a set alpha value of 0.05, the ability to achieve an effect size of 0.4 with 96.57% power with the sample in Study 1 indicated an adequate sample size.

#### 2.3.2. Experimental design

The experiment was a single-factor between-subjects design, with the independent variable being stereotype threat (yes/no) and the dependent variable being the rate of change in response time (ms) in the mental-rotation task during the experimental phase.

#### 2.3.3. Experimental procedures

Credamo’s HBO (behavioral experiments) template was used to create the main experimental procedures, which consisted of practice and formal experiments. Two practice trials were conducted before the formal start, and participants had to practice and confirm their understanding of the experimental rules before entering the formal experiment. The formal experimental task was divided into two phases. The first phase was used to obtain the participants’ baseline-level response times. The second phase was used to obtain the participants’ post-stimulus response times. The experimental material was a pattern involving the Chinese characters “Xi” and “Xi” (flipped horizontally) following six angles of rotation (30, 90, 150, 210, 270, and 330^°^).

**FIGURE 1:**
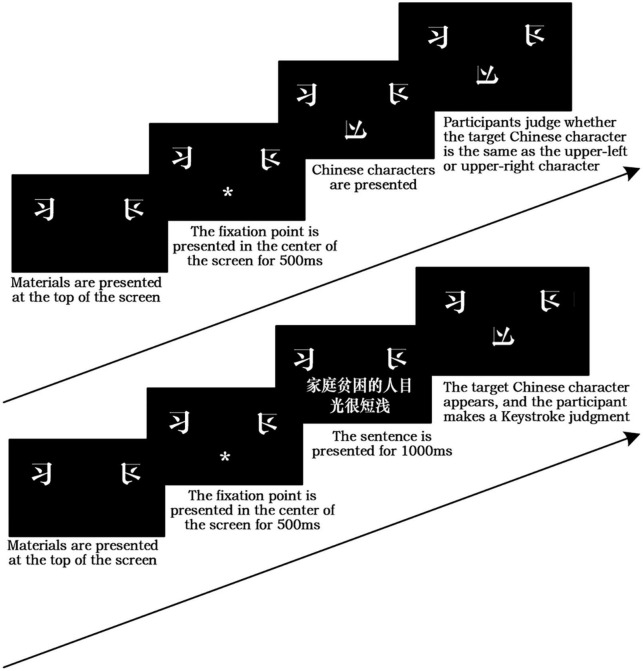
Schematic diagram of the experimental group’s mental rotation task. *Representing the reminder to the participant that stimulus sentences will appear in this location.

The experimental flow of the mental-rotation task is shown in Figure 1. Since the purpose of the first stage of the formal experimental task was to obtain the participants’ baseline-level response times, the mental-rotation task was identical for both groups, and the patterns of “Xi” and “Xi” (flipped) were displayed in the top left- and top right-hand corners of the screen. The participants had to decide whether the target pattern that appeared in the middle was derived from the rotation of “Xi” or “Xi” (flipped), and to respond by pressing a key. Specifically, the “F” key was pressed for “Xi” and the “J” key for “Xi” (flipped). In the second stage, the mental-rotation task remained the same. Before the start of each trial, however, a stimulus statement appeared on the screen. The experimental group saw statements incorporating the ten negative stereotypes mentioned above, while the control group saw neutral expressions of the same length. After the statements disappeared, a mental-rotation target pattern appeared and the participants continued to perform keystroke responses. The participants’ response times and accuracy rates were recorded. Following this, a number of demographic-related variables were collected from participants, including age, gender, and grade level.

The experiment was conducted in a secondary-school computer classroom in the participants’ own township to ensure a consistent research experimental environment. During the study, the temperature, light, and order of the experimental environment were well controlled; the examiner clarified that participants were not allowed to talk or walk around freely during the experiment. A psychology assistant was responsible for allocating seats and distributing materials. Before the experiment began, the examiner introduced the test to all participants as a reaction-speed-related keystroke-response test and explained the rules. After the participants had completed the mental-rotation task, the true purpose of the experiment was explained to them. The entire experiment lasted approximately 15–20 min.

#### 2.3.4. Analysis of results

To analyze the results and control for the effect of individual differences on the mental-rotation task, this study compared participant reaction times during the first and second phases of the formal experimental task (i.e., comparing the second-phase reaction time to the first-phase reaction time). The rate of change between reaction times differed significantly between the two groups of participants: [F(1,118) = 4.51, *p* < 0.01, η^2^ = 0.38]. For participants in the stereotype-threat group, reaction times increased after the stimulus (0.21); for those in the control group, reaction times decreased after the stimulus (–0. 17). The different statements presented to the two groups caused different reaction times between the two groups: participants who encountered negative stereotypes during the second stage had a longer second-phase reaction time, while participants who encountered neutral statements had a shorter second-phase reaction time, as illustrated in Table 2.

**TABLE 2:** Reaction times at different stages of the mental rotation task in both groups of participants.

M (SD)	Before stimulation (ms)	After stimulation (ms)
Stereotype-threat group	2506.39 (942.13)	2932.97 (1079.87)
Control group	2666.02 (1085.11)	2062.49 (769.73)

#### 2.3.5. Summary

Based on negative stereotypes associated with generationally poor individuals, Study 1 used a mental-rotation task to elicit and test the impact of stereotype threat and proved that this threat impacted the performance of generationally poor individuals in the mental-rotation task. In the experimental task, different phase-two statements led to a difference in phase-two reaction times between the two groups of participants. Participants in the stereotype-threat group had an increased reaction time, while participants in the control group had a decreased reaction time. Participants in the experimental group experienced a significant increase in both their psychological and cognitive loads, due to the activation of the stereotype-threat effect. In other words, the negative stereotypes presented to participants induced a stereotype threat that interfered with their ability to complete the mental-rotation task and prevented their phase-two reaction times from decreasing. By contrast, participants in the control group experienced a decrease in reaction time after being stimulated with neutral statements, possibly due to the emergence of a practice effect, i.e., a decrease in reaction-time levels following a few repeated trials, which made the participants more proficient in the experimental procedure.

## 3. Study 2: Impact of stereotype threat on endogenous poverty-elimination dynamics

### 3.1. Purpose of the study

First, the extent to which the mental-rotation task effectively induced and activated stereotype threat was verified through repeated use of the mental-rotation task. Second, based on the successful and effective introduction of stereotype threat, the present study was able to explore its effect on endogenous poverty-elimination dynamics among generationally poor individuals.

### 3.2. Research hypothesis

The stereotype-threat group experienced lower-level endogenous dynamics for poverty elimination than the control group, which was not subjected to stereotype threat.

### 3.3. Research methods

#### 3.3.1. Participants

Sixty generationally poor individuals were selected from a rural area in Hainan Province and invited to participate in the trial after providing their informed consent. There were 34 males and 26 females with a mean age of 31.89 years (SD = 1.23). All participants were randomly assigned to either the stereotype-threat group (12 males and 18 females) or the control group (22 males and 8 females).

#### 3.3.2. Experimental design

A single-factor between-subjects design was used for the experiment. The independent variable was stereotype threat (yes/no), while the dependent variable was the rate of change in participants’ mental rotation responses at the experimental-task stage, as well as their scores on the endogenous dynamics of poverty-elimination scale.

#### 3.3.3. Experimental procedure

Stereotype-threat elicitation: Based on the mental-rotation task, combined with the method of eliciting negative stereotypes used in the previous study. The same elicitation method has been employed in different studies, where the reproducibility and validity of the method can be examined.

Measurement of the endogenous dynamics of poverty alleviation: the endogenous-dynamics scale of poverty elimination developed by Wu et al. (2021) was used to assess endogenous poverty-elimination dynamics among individuals living with generational poverty. The scale includes three categories: values, self-perception, and the behavioral tendency to escape poverty (among individuals living with generational poverty). It contains a total of 20 items, scored using a five-point scale, with responses ranging from 1 (“do not conform/agree at all”) to 5 (“completely conform/agree”); the higher the total score, the stronger the endogenous poverty-elimination dynamics among individuals. The overall Cronbach’s alpha coefficient was 0.94 for the endogenous-dynamics scale of poverty elimination used in this study. The Cronbach’s alpha coefficients of the three categories of values, self-perception, and the behavioral tendency to escape poverty were 0.89, 0.82, and 0.82, respectively.

The present study was conducted in the same computer classroom as Study 1. To ensure a consistent research experimental environment, the researcher controlled the temperature, light, and order of the experimental environment; the main examiner also informed participants that they were not allowed to talk or walk around freely during the experiment. A psychology assistant was responsible for allocating seats and distributing materials. After randomly assigning all participants to one of two conditions, the main examiner introduced the test as a test of reaction speed and explained the rules. After the participants had completed the mental-rotation task, they completed the endogenous-dynamics scale of poverty elimination and added demographic variables, such as gender and age. At the end of the experiment, the main examiner explained clearly to participants the real purpose of the experiment. Each experimental session has a duration of approximately 15 to 20 min. The complete self-affirmation study spans a period of 10 days, during which participants in the experimental group are instructed to engage in self-affirmation once daily, while those in the control group are asked to narrate their daily experiences. The experimenter records daily progress throughout the study period.

#### 3.3.4. Analysis of results

A comparison of the reaction times of the two groups of participants revealed a significant between-group difference, [F(1,58) = 35.55, *p* < 0.01, η^2^ = 0.37], with an increased reaction time in the stereotype-threat group (0.18) and a decreased reaction time in the control group (–0.20), as illustrated in Table 3. This proved that the stereotype-threat elicitation was effective.

**TABLE 3:** Reaction times at different stages of the mental rotation task in both groups of participants.

M (SD)	Before stimulation (ms)	After stimulation (ms)
Stereotype-threat group	2537.59 (861.83)	2995.87 (1271.72)
Control group	2913.82 (1036.76)	2282.81 (787.52)

The results of the data analysis of the endogenous dynamics of poverty-elimination scores showed little difference between the scores of the two groups of participants in the values category, [F(1,58) = 0.17, *p* = 0.693 > 0.05]; similarly, their self-perception scores were roughly the same, [F(1,58) = 3.86, *p* = 0.056 > 0.05]; in the behavioral-tendency-to-escape-poverty category, participants in the stereotype-threat group scored significantly lower than those in the control group, [F(1,58) = 4.38, *p* = 0.042 < 0.05, η^2^ = 0.07]. Overall, participants in the stereotype-threat group scored significantly lower than those in the control group on endogenous poverty-elimination dynamics, [F(1,58) = 4.198, *p* = 0.046 < 0. 05, η^2^ = 0.069], as illustrated in Table 4. The results of this study suggest that stereotype threat reduces endogenous poverty-elimination dynamics in generationally poor individuals, causing their behavioral-tendency-to-escape-from-poverty scores to fall. The results support the experimental hypothesis.

**TABLE 4:** Endogenous dynamics of poverty elimination in the two groups.

M (SD)	Total score for the endogenous dynamics of poverty elimination	Values	Self-perception	Behavioral tendency to escape poverty
Stereotype-threat group	3.81 (0.26)	4.06 (0.30)	3.71 (0.53)	3.45 (0.51)
Control group	3.98 (0.41)	4.11 (0.53)	4.01 (0.89)	3.71 (0.52)

#### 3.3.5. Summary

Stereotype threat makes people less motivated to perform well (Woodcock et al., 2012). In Study 2, stereotype-threat activation had a negative effect on endogenous poverty-elimination dynamics in generationally poor individuals. The life values of poor individuals are characterized by accepting fate, settling for the *status quo*, and drifting along (Fu et al., 2020). Such negative core values render endogenous poverty-elimination dynamics more susceptible to the negative effects of stereotype threat when people face a stereotype-threat situation. At the same time, poor individuals have low self-efficacy and are more likely to accept negative cues and to evaluate themselves negatively (Hadden et al., 2020). When situational cues include negative stereotypes, the resulting stereotype threat triggers negative self-assessment and self-integrity crises among generationally poor individuals, making them more vulnerable to the negative impact of stereotype threat on endogenous poverty-elimination dynamics.

## 4. Study 3 the impact of self-affirmation on endogenous poverty-elimination dynamics in generationally poor individuals

### 4.1. Purpose of the study

Based on the ability to mitigate stereotype threat through self-affirmation (Hadden et al., 2020), this study explored the intervention effect of self-affirmation by investigating differences caused by the presence or absence of self-affirmation in endogenous poverty-elimination dynamics among generationally poor individuals, following activation of the stereotype-threat effect.

### 4.2. Research hypothesis

After a stereotype threat was activated, the self-affirmation intervention effectively mitigated the negative effects of stereotype threat on generationally poor individuals. This made the endogenous poverty-elimination dynamics higher in the self-affirmation group than in the control group.

### 4.3. Research methods

#### 4.3.1. Participants

Sixty generationally poor individuals were selected from a rural area in Hainan Province and invited to participate in the trial after giving their informed consent. There were 24 males and 36 females with a mean age of 29.98 years (SD = 0.97). The participants were randomly assigned to either the self-affirmation group (10 males and 20 females) or to the control group (14 males and 16 females).

#### 4.3.2. Experimental design

The experiment had a single-factor between-subjects design, where the independent variable was self-affirmation (yes/no) and the dependent variable was the score on the endogenous-dynamics scale of poverty elimination.

#### 4.3.3. Experimental procedures

Like the previous study, this study elicited stereotype threat and measured endogenous poverty-elimination dynamics. The mental-rotation task, combined with negative stereotype descriptions, was used to elicit stereotype threat; the endogenous-dynamics scale of poverty elimination was used to measure the participants’ endogenous poverty-elimination dynamics. The endogenous-dynamics scale of poverty elimination used in this study was the same as that in Study 2, with a Cronbach’s alpha coefficient of 0.94 for the overall scale and 0.89, 0.82, and 0.82 for the three categories of values, self-perception, and behavioral tendency to escape poverty, respectively.

Self-affirmation was selected as a method previously used by Chinese researchers and easy for students to understand and implement (He, 2012); the participants achieved self-affirmation by evaluating their own interpersonal relationships and positive characteristics. Three questions asked participants to list and describe significant others in as much detail as possible and to recall pleasant experiences with them, e.g., “Please describe a past event that makes you feel proud and honored”; “Please think of a happy time with someone you are close to”; and “Please think of someone who has brought you growth.” The other three questions asked participants to list and describe in detail as many of their own important and positive characteristics as possible: “Please recall one thing you have successfully accomplished”; “Please think of one or two of your own strengths or virtues”; and “Please give yourself three compliments.” In the control group, participants were asked to recall and describe their experiences of the day: “Please recall what you did this morning”; “Please describe what you have eaten over the past 24 h (by type or time).”

The experiment was conducted in a secondary-school computer classroom in the township where the research was carried out. First, all participants were subjected to stereotype-threat elicitation. Prior to the start of the mental-rotation task, the test was introduced to all participants as a response-speed test and the rules were explained. Next, all participants were randomly assigned to either the self-affirmation group or the control group, with one group answering self-affirmation questions and the other group daily-experience questions. When performing the self-affirmation, participants were asked to consider their own values carefully; this generally involved considering their own self-worth for 5–10 min, a more in-depth information-processing experience. Both groups of participants responded within the same time limit, after which their endogenous poverty-elimination dynamics were measured. At the end of the experiment, the main examiner explained the real purpose of the experiment and thanked the participants. The whole experiment lasted 15–20 min.

#### 4.3.4. Analysis of results

The analysis of endogenous poverty-elimination dynamics showed an insignificant difference between the scores of the two groups of participants in the self-perception category, [F(1,58) = 0.57, *p* = 0.458 > 0.05] and in the behavioral-tendency-to-escape-poverty category, [F(1, 58) = 0.08, *p* = 0.81 > 0.05]. In the value category, the self-affirmation-group scores were much higher than those of the control group, [F(1,58) = 23.02, *p* < 0. 01, η^2^ = 0.29]. Overall, participants in the self-affirmation group had significantly higher overall scores for the endogenous dynamics of poverty-elimination than those in the control group, [F(1,58) = 4.47, *p* = 0.041 < 0.05, η^2^ = 0.07], as illustrated in Table 5. This result also verifies the hypothesis.

**TABLE 5:** Endogenous dynamics scale for the poverty-elimination scores of both groups.

M (SD)	Total score for the endogenous dynamics of poverty elimination	Values	Self-perception	Behavioral tendency to escape poverty
Self-affirmation group	3.89 (0.27)	4.01 (0.46)	3.92 (0.48)	3.62 (0.59)
Control group	3.63 (0.29)	3.52 (0.27)	3.84 (0.58)	3.56 (0.58)

#### 4.3.5. Summary

Following the stereotype threat, participants who performed a self-affirmation showed much more improvement in their endogenous poverty-elimination dynamics than those who did not engage in self-affirmation. Self-affirmation was able to buffer or reduce the psychological threat to participants and reduce their defensive adaptation to the psychological threat, allowing them to focus more on the psychological-rotation task itself, rather than making defensive preparations for the stereotype-threat stimulus. Thus, participants who engaged in self-affirmation were less hindered by the psychological threat and better able to organize their cognitive resources to meet the demands of the task at hand. Overall, the negative consequences of stereotype threat were attenuated through self-affirmation.

## 5. Discussion

### 5.1. Validity of stereotype-threat induction in generationally poor individuals

Mental-rotation tasks have often been used to investigate stereotype-threat activation; some studies have focused on working memory as an explanatory mechanism (Rydell et al., 2010). The present study has used negative stereotypes associated with generationally poor individuals to elicit and test the effects of stereotype threat *via* a mental-rotation task. The results show that stereotype threat affects the performance of generationally poor individuals in a mental-rotation task. In the experimental task, different stimulus statements were presented to two groups of participants. As a consequence, the control-group participants had a shorter reaction time during the second stage, while the stereotype-threat participants had a longer reaction time during the second stage after being stimulated with negative stereotypes. This can be explained using the integrated-process model proposed by Schmader et al. (2008), where stereotype-threat messages in the experiment caused generationally poor individuals to feel pressured and to consciously monitor their behavioral performance and to suppress the resulting negative emotions. As these processes occupied a large amount of working memory, individuals had insufficient resources left to deal with complex cognitive tasks and thus performed less well on the mental-rotation tasks.

### 5.2. Effects of stereotype threat on endogenous poverty-elimination dynamics in generationally poor individuals

Stereotype threat can easily trigger psychological disidentification from a domain, reducing the motivation to perform better in that domain (Woodcock et al., 2012). In the present study, stereotype-threat activation had a negative effect on endogenous poverty-elimination dynamics among generationally poor individuals; the reduced endogenous poverty-elimination dynamics made them more likely to conform to negative stereotypes through their behavior. First, among the three main domains of endogenous poverty-elimination dynamics, the life values of poor individuals are specifically expressed as accepting fate, settling for the *status quo*, and drifting along (Fu et al., 2020). When people face a stereotype-threat situation, these negative core values undermine the intrinsic driving force that guides behavior designed to escape poverty. Second, in the self-perception category, poor individuals are characterized by low self-efficacy, accepting negative cues, and evaluating themselves negatively (Fu et al., 2020). When situational cues include negative stereotypes, the resulting stereotype threat triggers negative self-assessment and self-integrity crises among generationally poor individuals. These negative self-perceptions work together to validate the negative stereotypes in question. Finally, in stereotype-threat situations, individuals living with generational poverty lack strategies for coping with daily life tasks, goal setting, personal planning, and financial investment, due to their relatively low behavioral tendency to escape poverty (Fu et al., 2020); this further validates the corresponding negative stereotypes.

### 5.3. Intervening role of self-affirmation

Self-affirmation serves as an intervention tool to enhance self-value and increase positive self-concept, thereby mitigating the negative impact of stereotype threat. With regards to emotional experience, self-affirmation boosts an individual’s sense of self-worth, providing a sense of security that “I am valuable” (Sherman and Kim, 2005). In terms of psychological coping, self-affirmation functions as a buffer against the impact of threatening information on self-value, reducing the challenging nature of the information, lowering the individual’s defense level, and making them more receptive to threatening information (Sherman and Cohen, 2002; Correll et al., 2004). Stereotype threat can undermine an individual’s self-integrity; events are threatening precisely because of their impact on self-integrity (Silverman and Cohen, 2014). When confronted with a specific threat, people can access psychosocial resources that transcend the specific threat and broaden their perspective, helping them assess the threat *via* self-affirmation in areas unrelated to the specific threat domain. This enables them to improve their performance in the negative-stereotype domain (Zhang et al., 2014). Individuals who engage in self-affirmation interventions are less hindered by psychological threats and better at organizing their cognitive resources to meet the demands of the task at hand. At the same time, self-affirming participants also pay more attention to the errors they make in cognitive tasks and are more willing to learn from their errors, rather than focusing on defensive preparations (Sherman and Cohen, 2006). In this study, the generationally poor individuals who engaged in self-affirmation had higher endogenous poverty-elimination dynamics than those who did not engage in self-affirmation, after being threatened by negative stereotypes. The self-affirmation intervention strategy is a series of exercises that enable people to demonstrate their adequacy or reflect their core personal values. Second, self-affirmation triggered self-evaluation changes, which in turn affected self-perceptions, such as self-efficacy and self-regulation. For this reason, high levels of self-affirmation mitigate the negative effects of stereotype threats. Finally, participants reduced their fear of threats through self-affirmation, focusing instead on confronting and solving problems. They were thus more likely to think of better strategies for escaping poverty; they also had a higher propensity toward poverty-eradicating behaviors. This study combines an analysis of three categories of endogenous poverty-elimination dynamics among generationally poor individuals, showing that self-affirmation as an intervention can effectively mitigate the negative effects of stereotype threat.

### 5.4. Theoretical and practical implications

In its theoretical implications, this study enriches the psychological basis of poverty alleviation. At each decision-making point, the choice outcomes of poor individuals are influenced by both external and psychological resources, resulting from a combination of external and psychological resources (Fu and Huang, 2018). Of these, the external resources include natural, economic, social (interpersonal), family, educational, and psychological resources, including endogenous motivation. The combined lack of internal and external resources creates the difficulties that poor individuals face in trying to escape poverty. Although previous studies of poverty alleviation have focused on external resources, it is more important to help poor individuals alleviate poverty. Poor individuals lack endogenous poverty-elimination dynamics, a core psychological resource for eliminating poverty (Fu et al., 2020). Unlike previous family-related perspectives, this research on stereotype threats among generationally poor individuals provides a new perspective on the factors that affect endogenous poverty-elimination dynamics. Such factors are referred to as a “threat in the air,” due to their universality and widespread and pervasive nature in everyday life (Steele, 1997). In discussing the impact of stereotype threat on individuals living with generational poverty, this study explores the impact of stereotype threats on endogenous poverty-elimination dynamics from the perspective of internal psychological resources—a perspective that has not been previously explored in the literature. This research therefore provides a more comprehensive understanding of the causes of endogenous poverty-elimination dynamics, providing practical and effective ideas and strategies for addressing related problems and enriching research in the field of stereotype threats and endogenous poverty-elimination dynamics.

In terms of practical implications, self-affirmation provides a simple and feasible intervention for psychological poverty alleviation. This study has examined the effectiveness of a self-affirmation intervention to combat stereotype threat through an experimental design, providing ideas for the practice of psychological poverty-alleviation, i.e., intervening with generationally poor individuals through self-affirmation to enhance their endogenous poverty-elimination dynamics. In addition, compared to other stereotype-threat interventions, the self-affirmation intervention used in this study is simple and easy to understand. It can be introduced easily into real-life situations as well as being used in experimental studies.

### 5.5. Research limitations and future prospects

First, as participants came from a specific group, the sample selection had some limitations. Future studies should further explore the effectiveness of stereotype-threat elicitation methods by expanding the sample size or extending the study population. Second, this study is geared toward a short-term stereotype threat in a specific context. Future studies can further distinguish between the immediate and delayed effects of stereotype threat. Third, previous studies of stereotype-threat interventions have covered many other intervention strategies, including enhancing self-efficacy; future studies should consider other types of intervention strategies and explore their effectiveness. Finally, This study utilized laboratory methods to conduct research, which proficiently controlled for extraneous variables and bolstered the internal validity of the experiment. Nevertheless, this approach may engender a diminution in ecological validity. Hence, we intend to execute field experiments in actual environmental settings to further corroborate our research hypotheses.

## 6. Conclusion

This study explores the effects of stereotype threat on endogenous poverty-elimination dynamics in generationally poor individuals; it also investigates the effectiveness of a self-affirmation intervention. The results indicate that (1) stereotype threat can be elicited in generationally poor individuals and tested through a combination of relevant negative stereotypes and a mental-rotation task; (2) the presence of a stereotype threat reduces endogenous poverty-elimination dynamics in generationally poor individuals; and (3) self-affirmation interventions can mitigate the negative effects of a stereotype threat on endogenous poverty-elimination dynamics in generationally poor individuals.

## Data availability statement

The raw data supporting the conclusions of this article will be made available by the authors, without undue reservation.

## Ethics statement

The studies involving human participants were reviewed and approved by the Hainan University Institutional Review Board. The patients/participants provided their written informed consent to participate in this study.

## Author contributions

NW, AF, and YL contributed to the conception and the design of the work as well as the preparation of the draft. NW and TY contributed to the analysis and interpretation of the data. JL, XW, and XH critically reviewed and contributed important intellectual input. All authors contributed to the article and approved the submitted version.
